# Supplementary material for: Development of a long noncoding RNA-based machine learning model to predict COVID-19 in-hospital mortality
Source: Nat Commun. 2024 May 20;15:4259. doi: 10.1038/s41467-024-47557-1 (PMC11106268; doi:10.1038/s41467-024-47557-1)

Supplementary material

Supplementary methods

ISARIC4C study design, registration and approvals

Samples were received from the ISARIC WHO Clinical Characterization Protocol for Severe Emerging Infections in the UK (CCP-UK), an ongoing prospective cohort study of hospitalized patients with COVID-19, which is recruiting in 258 hospitals across England, Scotland, and Wales (National Institute for Health Research Clinical Research Network Central Portfolio Management System ID: 14152) (https://isaric4c.net/). This is a pre-approved pandemic preparedness study using a publicly available protocol with urgent public health research status. The ISARIC4C study collected comprehensive sets of data and samples from hospitalized patients with COVID-19 to facilitate better understanding of the disease process and to assist in developing effective treatments. The protocol, including revisions, case report forms, patient information leaflets, consent forms and details of the Independent Data and Material Access Committee are available online (https://isaric4c.net/).

The ISARIC WHO CCP-UK study was registered at https://www.isrctn.com/ISRCTN66726260 and designated an Urgent Public Health Research Study by the National Institute for Health Research UK. Ethical approval was given by the South Central - Oxford C Research Ethics Committee in England (Ref 13/SC/0149), the Scotland A Research Ethics Committee (Ref 20/SS/0028), and the WHO Ethics Review Committee (RPC571 and RPC572, 25 April 2013). Healthy controls were recruited specifically for the purpose of this study from healthy donors following informed consent at Newcastle University (REC reference 12/NE/0121).

Library preparation, targeted RNA sequencing and raw data analysis

A second stratified randomization by age and sex was performed in batches of 46 samples prior to library preparation. The KAPA Stranded RNAseq Kit with RiboErase (HMR; Cat. #634444, Roche diagnostics, Basel, Switzerland) was used for ribosomal RNA (rRNA) depletion and total RNA libraries construction. The libraries were generated by the EpMotion 5075t NGS solution (Cat: 5075000962, Eppendorf, Hamburg, Germany), an automated pipetting system. The starting RNA input quantity for each sample was set at 100 ng in a total volume of 10 µL. The library preparation started with the removal of cytoplasmic and mitochondrial rRNA, followed by a DNase digestion to eliminate any genomic DNA that might be present in the sample. RNA was fragmented by heat digestion and magnesium ions, and was then retrotranscribed to cDNA by reverse transcription using random priming. The first and second strands cDNA were synthesized followed by a post strands synthesis clean-up performed using Celemag clean-up beads (Cat. #CMCB57.6, Celemics, Seoul, Korea). On-beads cDNA samples were left overnight in the A-Tailing buffer. The next day, double stranded cDNA fragments were A-tailed by adding a dAMP at the 3'-end of each fragment, followed by the adapter ligation. After ligation, two successive clean-ups were performed using a PEG/NaCl solution to select the size of the cDNA libraries and remove adapter dimers. Finally, purified ligated cDNA samples were amplified by a 13-cycle low-bias PCR. During the PCR, cDNA fragments were dual indexed using the library preparation box #2 (Cat. # LI20D96, Celemics, Seoul, Korea) allowing them to be identified after sequencing.

The indexed libraries were then captured using FIMICS (Cat. #BO5096, Celemics, Seoul, Korea), a probe panel targeting 3233 cardiac-enriched or heart failure-associated lncRNAs 7. The libraries were hybridized to the biotinylated target FIMICS capture probes for 24 hours. Captured lncRNA sequences were then purified using Celemag streptavidin coated magnetic beads (Cat. #CMSB5.76, Celemics, Seoul, Korea). Six successive heated washes using Celemics wash buffer (Cat. #TC4096, Celemics, Seoul, Korea) were performed, with a 10-minute incubation at 65°C to eliminate non-specific binding from the beads. Finally, the on-beads captured sequences were enriched by PCR (14 cycles) and PCR products were purified using Celemag clean-up beads.

The enriched libraries were quantified using the Qubit 3.0 fluorometer with the 1X dsDNA high sensitivity Assay kit, and their quality was assessed using the TapeStation 4150 system (Cat. #G2992AA, Agilent, Santa Clara, CA, USA). The HS D1000 ScreenTape associated with the HS D1000 Reagents were used to assess the quality of the samples. Absence of adapter dimers was checked, and the average library size was determined by a region table.

The libraries were then sequenced on the NextSeq 2000 platform (Cat. #20038897, Illumina Inc., San Diego, CA, USA). Before each run, all samples were normalized and pooled in equimolar concentrations. The NextSeq 2000 denatured and diluted the pooled libraries on board prior to sequencing. The NextSeq 2000 P2 kit (Cat. #20046811, Illumina Inc., San Diego, CA, USA) containing a reagent cartridge (100 cycles), and a P2 flow cell was used for each run. P2 flow cells have a reads passing filter of 400 M reads which allowed the simultaneous quantification of 48 samples. In average, 8 million reads were yielded per sample. Libraries were sequenced in paired end (2x50bp). PhiX Control (Cat. #15041963, Illumina Inc., San Diego, CA, USA) was added to the samples before each sequencing run to add diversity in each pool and to use it as a spike-in control.

Raw sequencing data were analysed using the Firalink pipeline organized in five steps [8]. The first step is the quality control of the FASTQ files using the FastQC software. The second step uses the Trimmomatic software [33] to remove low-quality reads with Phred score below 30. The third step evaluates potential contaminations in each sample using Kraken, a taxonomic classification tool used to detect inter-species contaminations. The fourth step is the alignment of the reads on the hg19 human genome, the generation of count tables for each sample and the quantification of lncRNAs by comparing the count tables to the FIMICS panel. All this process is performed by Kallisto software. The fifth and last step is the compilation of quality control (QC) files using MultiQC tool that compiles all the above information and creates a QA/QC report.

Table S1. Comparisons of characteristics of patients from the three EU cohorts. The p values for continuous variables are from 2-sided Student's t test. The p values for categorical variables are from 2-sided Fisher exact test.

	PrediCOVID (n=136)				NAPKON (n=556)				ISARIC4C (n=112)				*p* PrediCOVID vs. NAPKON vs. ISARIC4C*
	All	Non-survivors	Survivors	*P* non-survivors vs. survivors	All	Non-survivors	Survivors	*P* non-survivors vs. survivors	All	Non-survivors	Survivors	*P* non-survivors vs. survivors	
**Age (years, mean [SD])**	41[13]	-	41[13]	-	56[16]	70[12]	54[16]	5.7E-10	58[15]	66[15]	57[14]	3.1E-04	2.0E-22
**Male (n[%])**	73[53.7]	-	73[53.7]	-	330[59.4]	28[67.7]	302[58.8]	3.3E-01	77[68.8]	18[90]	59[64.1]	3.2E-02	5.2E-02
**Current smoker (n[%])**	29[21.3]	-	29[21.3]	-	40[7.2]	3[7.1]	37[7.2]	5.1E-01	4[3.5]	0[0]	4[3.5]	7.0E-01	7.9E-05
**Former smoker (n[%])**	22[16.2]	-	22[16.2]	-	153[27.5]	14[33.3]	139[26.8]	5.1E-01	30[26.8]	4[3.6]	26[23.2]	7.0E-01	4.5E-05
**Diabetes (n[%])**	7[5.15]	-	7[5.15]	-	124[22.59]	17[40.48]	107[21.10]	6.6E-03	21(20.00]	16(18.60]	5(26.32]	5.3E-01	2.8E-05
**Chronic lung disease (n[%])**	7[5.15]	-	7[5.15]	-	98[17.85]	16[39.02]	82[16.14]	9.3E-04	31(29.25]	29(33.33]	2(10.53]	5.5E-02	1.1E-05
**Cardiovascular disease (n[%])**	7[5.15]	-	7[5.15]	-	268[48.64]	34[80.95]	234[45.97]	9.5E-06	23(23.23]	18(22.22]	5(27.78]	7.6E-01	2.2E-16
**Cancer (n[%])**					73[13.13]	12[30.00]	61[11.82]	6.0E-03					
**Oxygen therapy (n[%])**	0[0]	-	0[0]	-	355[64.08]	39[92.86]	316[61.72]	1.6E-05	76(74.51]	59(71.08]	17(89.47]	1.4E-01	2.2E-16
**LDH (U/l, median[IQR])**					279[181]	284[166]	277[184]	6.0E-01					

**CRP (mg/l, median[IQR])**					45.1[85.2]	75.6[104.08]	44[85.8]	2.1E-03						
**COVID-19 vaccination**					166[39.5]	3[11.5]	163[41.4]	3.0E-03						
**Ethnicity**														
Caucasian (n[%])					414[87.90]	34[89.47]	380[87.76]	1.0E+00						
African (n[%])					7[1.49]	0[0.00]	7[1.62]	1.0E+00						
Asian (n[%])					10[2.12]	0[0.00]	10[2.31]	1.0E+00						
Arabic (n[%])					14[2.97]	2[5.26]	12[2.77]	3.1E-01						
Latin american (n[%])					1[0.21]	0[0.00]	1[0.23]	1.0E+00						
Mixed (n[%])					2[0.42]	0[0.00]	2[0.46]	1.0E+00						
Other (n[%])					23[4.88]	2[5.26]	21[4.85]	7.1E-01						
Common COVID-19 Symptoms														
Fever (n[%])	49[36]	-	49[36]	-	356[64.03]	333[59.9]	23[4.1]	2.2E-16	71[63.4]	61[54.5]	10[8.9]	2.2E-16	1.23E-08
Headache (n[%])	35[25.7]	-	35[25.7]	-	214[38.49]	205[36.87]	9[1.6]	2.2E-16	21[18.8]	20[17.9]	1[0.9]	2.7E-08	3.03E-05
Cough (n[%])	47[34.6]	-	47[34.6]	-	371[66.73]	351[63.13]	20[3.6]	2.2E-16	80[71.4]	67[59.8]	13[11.6]	2.2E-16	3.20E-12
Dyspnea (n[%])	12[8.82]	-	12[8.82]	-	323[58.09]	292[52.52]	31[5.57]	2.2E-16	72[64.3]	61[54.5]	11[9.8]	3.2E-16	2.2E-16

**p* value from ANOVA is shown.

Table S2. Characteristics of NAPKON patients used for PCR assessment. The p values for continuous variables are from 2-sided Student's t test. The p values for categorical variables are from 2-sided Fisher exact test.

	**NAPKON cohort**			
	**All (n=84)**	**Non-survivors (n=42)**	**Survivors (n=42)**	***P*** **non-survivors vs. survivors**
**Age (Years mean [SD])**	70[12.4]	70[12.3]	70[12.4]	0.88
**Male (n[%])**	55[65.5]	27[32.1]	28[33.3]	1.00
**Current smoker (n[%])**	4[4.8]	2[2.4]	2[2.4]	1.00
**Former smoker (n[%])**	20[23.8]	10[11.9]	10[11.9]	1.00
**Diabetes (n[%])**	33[39.28]	18[21.43]	15[17.86]	0.62
**Chronic lung disease (n[%])**	29[34.52]	16[19.05]	13[15.48]	0.60
**Cardiovascular disease (n[%])**	67[79.76]	35[41.66]	32[38.10]	0.73
**Cancer (n[%])**	16[19.05]	10 [11.90]	6 [7.14]	0.29
**Oxygen therapy (n[%])**	70[83.33]	40[47.62]	30[35.71]	0.13
**LDH (U/l, median[IQR])**	293[163.75]	290[148.5]	299.5[163.5]	0.49
**CRP (mg/l, median[IQR])**	32.70[75.43]	55.95[76.93]	14.55[57.48]	0.04

Table S3. Performance of different classifiers to predict in-hospital mortality in the discovery cohort using the two selected features (age and LEF1-AS1) without missing data imputation.

Classifier	AUC	Accuracy	Sensitivity	Specificity	Brier score
**RF**	0.81(0.79-0.82)	0.74(0.72-0.75)	0.75(0.72-0.77)	0.73(0.7-0.75)	0.18(0.17-0.19)
**kNN**	0.82(0.8-0.84)	0.76(0.74-0.78)	0.84(0.83-0.86)	0.68(0.65-0.7)	0.17(0.16-0.18)
**Logit**	0.82(0.8-0.84)	0.77(0.75-0.79)	0.82(0.8-0.85)	0.72(0.69-0.75)	0.17(0.16-0.18)
**MLP**	0.83(0.81-0.85)	0.78(0.76-0.8)	0.81(0.79-0.84)	0.75(0.72-0.77)	0.17(0.16-0.18)
**SVM**	0.69(0.64-0.74)	0.76(0.75-0.78)	0.82(0.8-0.84)	0.7(0.67-0.73)	0.2(0.19-0.21)
**XGB**	0.76(0.74-0.78)	0.69(0.67-0.71)	0.7(0.67-0.73)	0.68(0.65-0.71)	0.24(0.22-0.25)

Supplementary Figure 1. Model selection and evaluation procedure using machine learning. A. Model selection and evaluation on the balanced datasets. B. Model evaluation on the imbalanced datasets.

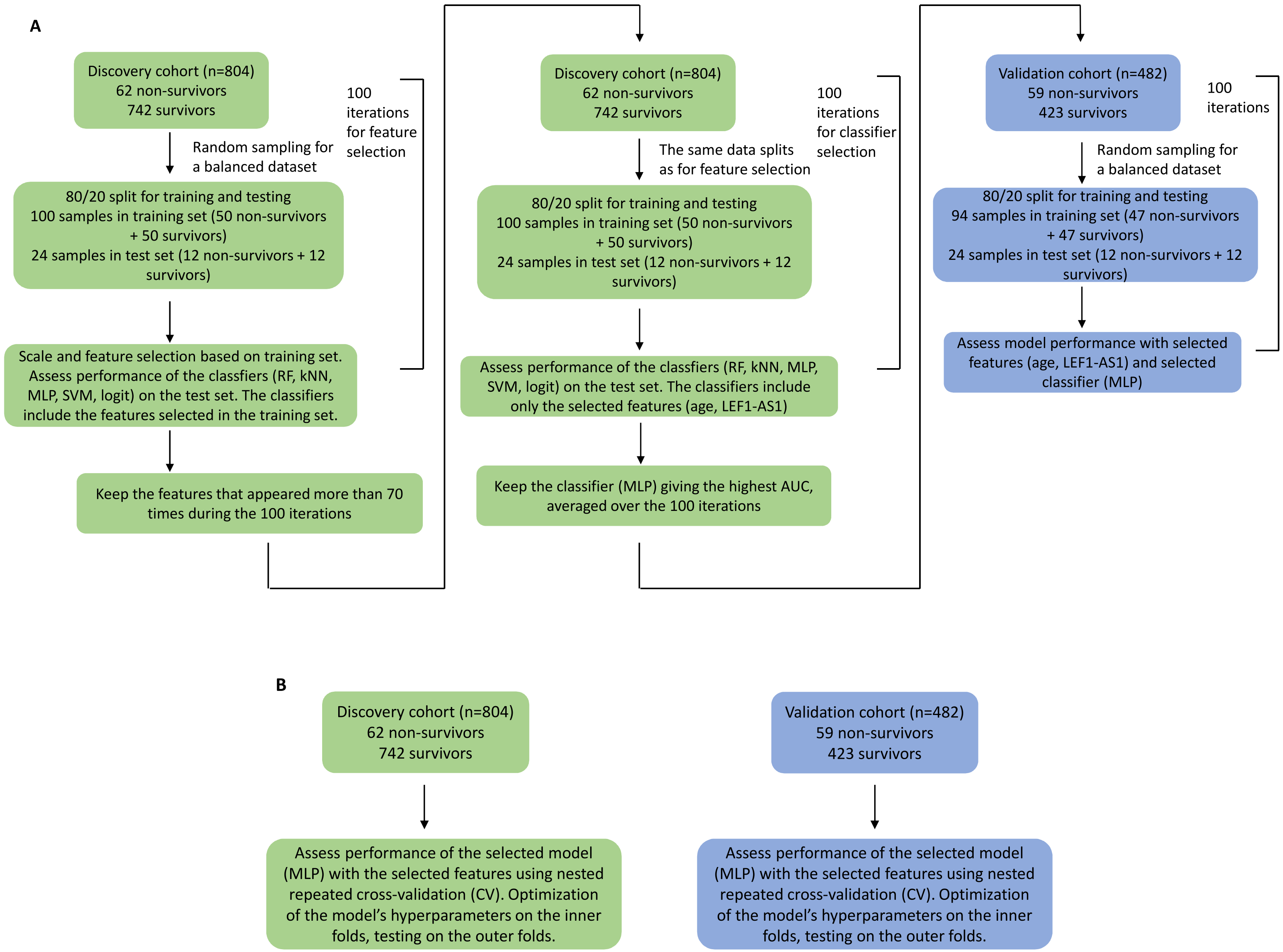

Supplementary Figure 2. Shapley beeswarm plots for the best predictive features (age and LEF1-AS1) in the Discovery and Validation cohorts.

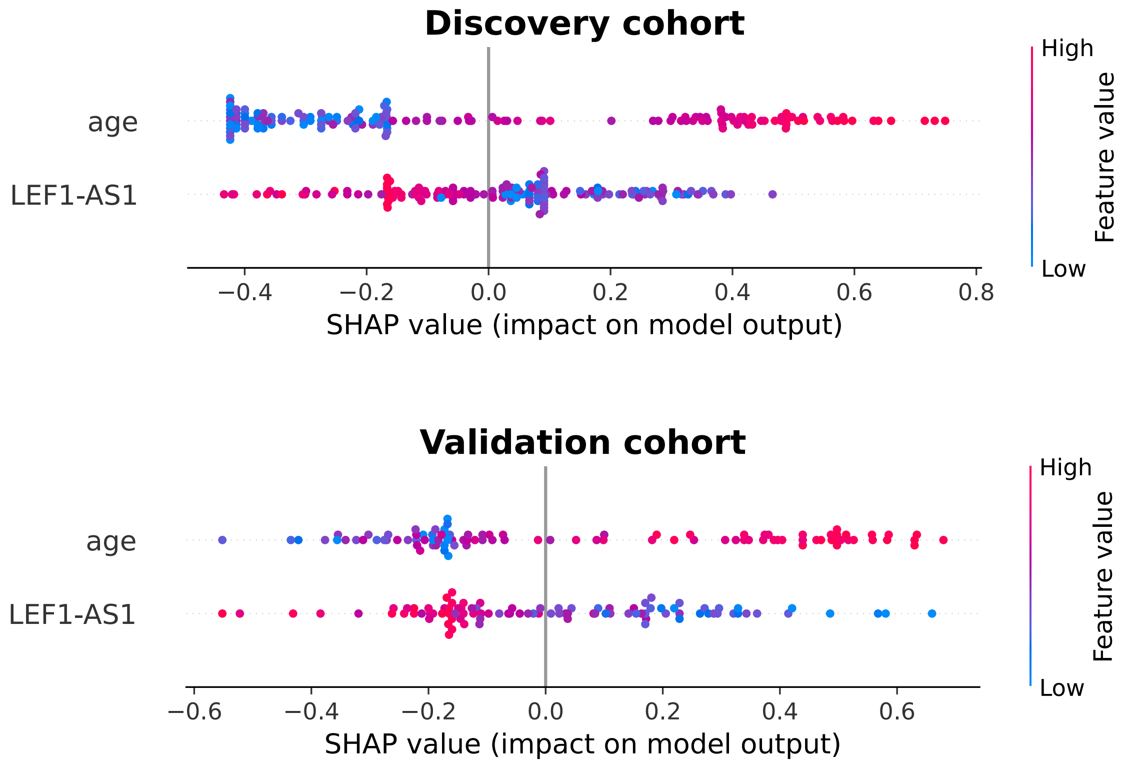

Supplementary Figure 3. Receiver operating characteristic curves of the MLP model training and evaluation using the discovery (n=804) and the validation data (n=482), respectively. The evaluation was performed on the balanced data from 100-iteration random sampling, and on the imbalanced data with 20 repeated 5-fold cross-validation.

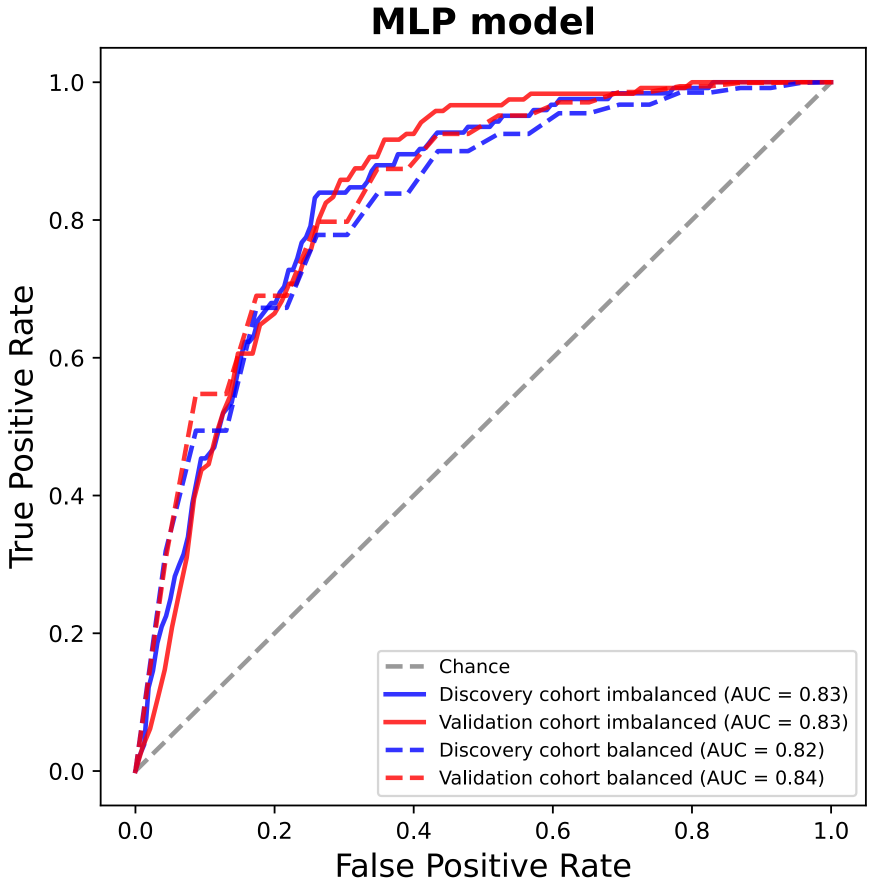

Supplementary Figure 4. Comparison of the performance of the models with age alone, LEF1-AS1 alone and the two features using the discovery (n=804) and the validation cohort (n=482), respectively. The evaluation was performed on the balanced data within 100 iterations. P values are from a two-sided student's t test.

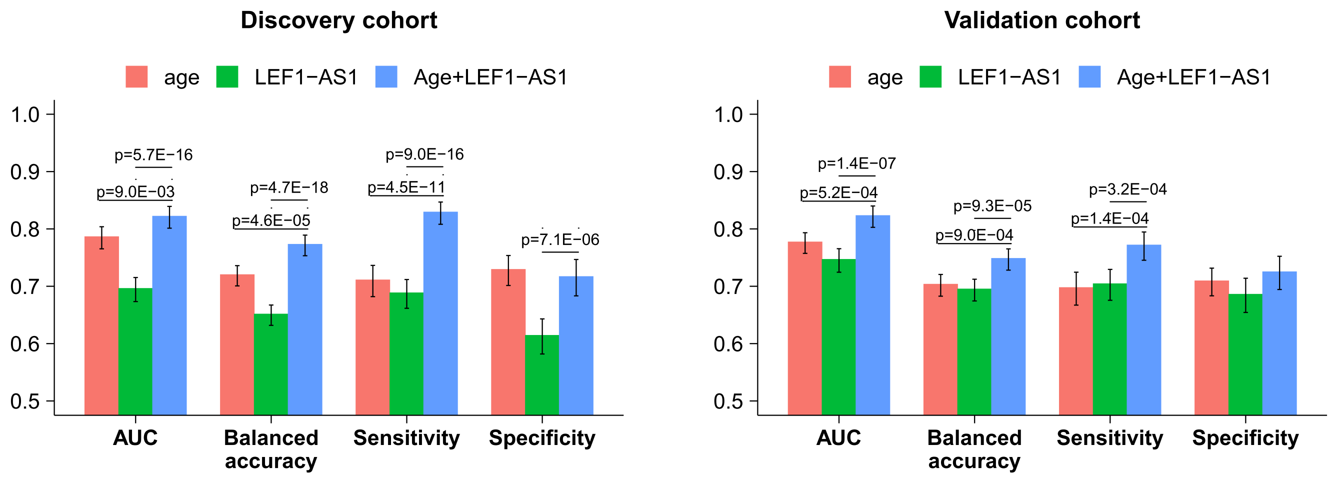

Supplementary Figure 5. MLP model performance after adding different combinations of sex and the other 2 features, which were selected more than 40 times in the feature selection iterations: oxygen therapy and SEQ0986.

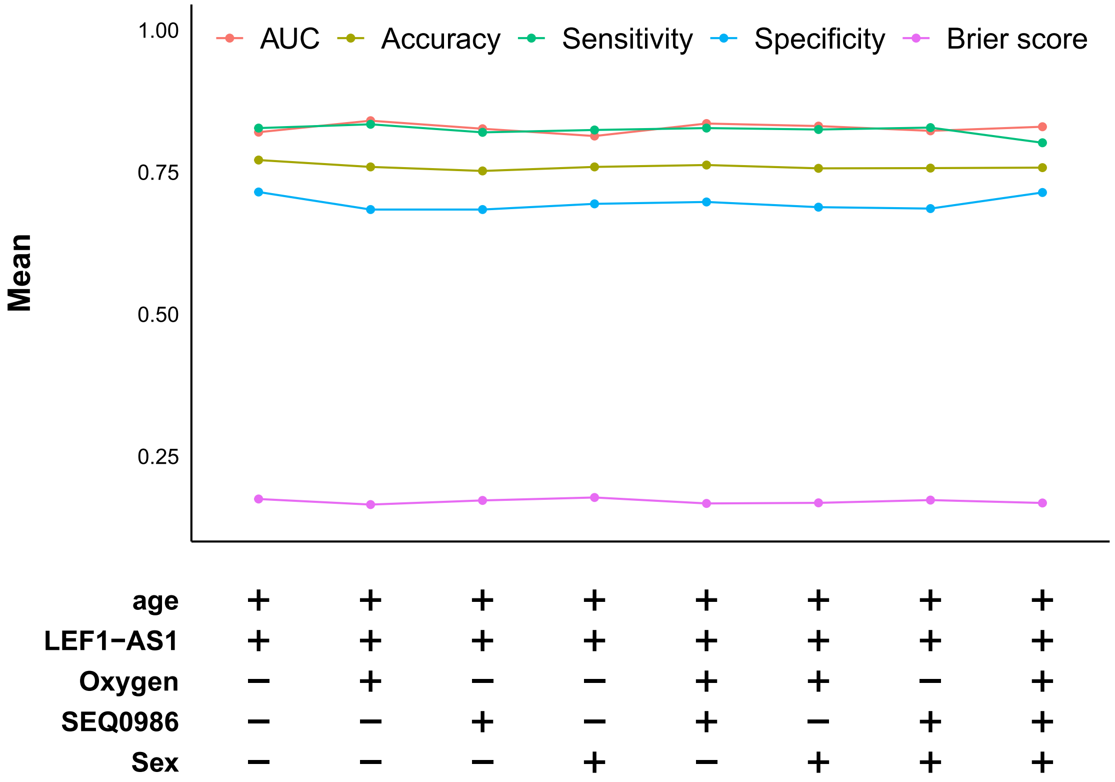

Supplementary Figure 6. Comparison of the predictive capacity of a previously established model (using age, sex, C-reactive protein (CRP) and lactate dehydrogenase (LDH)) versus our two features model (age and LEF1-AS1) in NAPKON and BCQ19 cohorts.

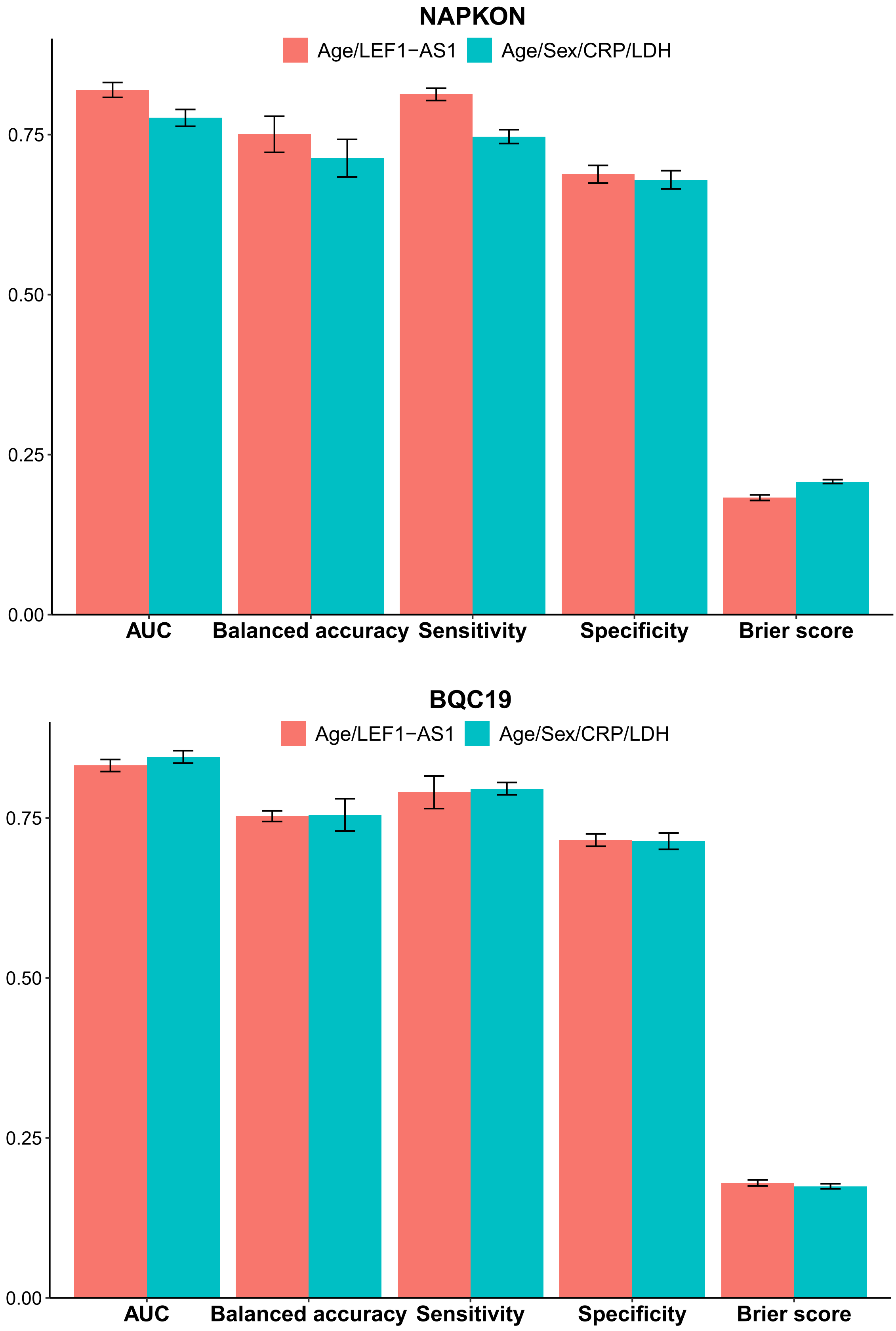

Supplement: Supplementary file 1 — Supplementary Information [file 41467_2024_47557_MOESM1_ESM.pdf]
